# A201 USTEKINUMAB VERSUS VEDOLIZUMAB PREDICTORS OF RESPONSE IN IBD PATIENTS WITH ANTI-TNF EXPOSURE: A PRELIMINARY REPORT

**DOI:** 10.1093/jcag/gwae059.201

**Published:** 2025-02-10

**Authors:** H Homsi, U Khan, B Lawendy, U Abbas, R Khanna, A Wilson

**Affiliations:** Schulich School of Medicine & Dentistry, Western University, London, ON, Canada; Schulich School of Medicine & Dentistry, Western University, London, ON, Canada; Schulich School of Medicine & Dentistry, Western University, London, ON, Canada; Schulich School of Medicine & Dentistry, Western University, London, ON, Canada; Schulich School of Medicine & Dentistry, Western University, London, ON, Canada; Schulich School of Medicine & Dentistry, Western University, London, ON, Canada

## Abstract

**Background:**

Ustekinumab and vedolizumab are biologics that are effective in treating inflammatory bowel disease (IBD). Previous studies using propensity score matching have found no significant difference in their efficacy overall. Interestingly, these studies did not highlight clinical variables associated with optimal drug response and other treatment outcomes which could help inform treatment selection for patients after anti-tumor necrosis factor (anti-TNF) agent exposure.

**Aims:**

To evaluate clinical variables associated with response to ustekinumab versus vedolizumab in IBD patients with anti-TNF exposure.

**Methods:**

A single-center retrospective cohort study was conducted in IBD patients with prior anti-TNF exposure, treated with one of ustekinumab or vedolizumab. Participants were assessed for clinical remission at 12 months using the Harvey Bradshaw Index for Crohn’s Disease (CD) (HBI≤4) and the Partial Mayo Score for Ulcerative Colitis (UC) (≤2). Secondary outcomes included 6-month remission, surgery, hospitalization, use of rescue glucocorticoids, adverse events, and treatment discontinuation in patients followed for at least 1 year. Potential clinical variables associated with the primary and secondary outcomes were assessed using multivariable logistic regression analyses for each drug group.

**Results:**

A total of 118 patients (CD, n=61; UC, n=57) treated at a tertiary care center in London, Ontario, Canada, were included in the analysis. Patients received at least one dose of ustekinumab (n=58) or vedolizumab (n=60). For both the vedolizumab-exposed and ustekinumab-exposed cohorts, 6- and 12-month disease remission were associated with high dose therapy (vedolizumab, 12-month, adjusted OR=6.62, 95%CI 1.95-26, p=0.004; 6-month, adjusted OR=4.77, 95%CI 1.43-17.58, p=0.014 versus ustekinumab, 12-month, adjusted OR=4.08, 95%CI 1.19-15.26, p=0.029; 6-month, adjusted OR=4.57, 95%CI 1.30-18.34, p=0.022). For the vedolizumab-exposed cohort, treatment discontinuation was associated with age (p=0.039), weight (p=0.031) and high dose therapy (p=0.008). Similarly, the need for rescue glucocorticoids was associated with age (p=0.021). No other clinical variables were associated with the primary or secondary outcomes for either cohort.

**Conclusions:**

Ustekinumab and vedolizumab demonstrated similar efficacy in achieving clinical remission in our study population. The odds of achieving remission from either at 6 and 12 months was higher amongst individuals receiving high-dose therapy. Vedolizumab treatment discontinuation was associated with a younger age and lower weight. No other clinical variables were associated with drug response in either cohort. Larger studies are warranted to confirm these findings and identify other predictors of response that can guide biologic selection in IBD management.

**Funding Agencies:**

None

